# Angiogenic gene expression and vascular density are reflected in ultrasonographic features of synovitis in early rheumatoid arthritis: an observational study

**DOI:** 10.1186/s13075-015-0567-8

**Published:** 2015-03-13

**Authors:** Stephen Kelly, Michele Bombardieri, Frances Humby, Nora Ng, Alessandra Marrelli, Sudeh Riahi, Maria DiCicco, Arti Mahto, Lu Zou, Debasish Pyne, Rebecca E Hands, Costantino Pitzalis

**Affiliations:** Rheumatology Department, Barts Health NHS trust Mile End hospital, London, EH1 4DG UK; Queen Mary University of London, Centre for Experimental Medicine and Rheumatology, William Harvey Research Institute, Charterhouse Square, London, EC1M 6BQ UK

## Abstract

**Introduction:**

Neovascularization contributes to the development of sustained synovial inflammation in the early stages of Rheumatoid Arthritis. Ultrasound (US) provides an indirect method of assessing synovial blood flow and has been shown to correlate with clinical disease activity in patients with Rheumatoid Arthritis. This study examines the relationship of US determined synovitis with synovial vascularity, angiogenic / lymphangiogenic factors and cellular mediators of inflammation in a cohort of patients with early Rheumatoid Arthritis (RA) patients prior to therapeutic intervention with disease modifying therapy or corticosteroids.

**Methods:**

An ultrasound guided synovial biopsy of the supra-patella pouch was performed in 12 patients with early RA prior to treatment. Clinical, US and biochemical assessments were undertaken prior to the procedure. Ultrasound images and histological samples were obtained from the supra-patella pouch. Histological samples were stained for Factor VIII and a-SMA (a-smooth muscle actin). Using digital imaging analysis a vascular area score was recorded. QT-PCR (quantitative-PCR) of samples provided quantification of angiogenic and lymphangiogenic gene expression and immunohistochemistry stained tissue was scored for macrophage, T cell and B cell infiltration using an existing semi-quantitative score.

**Results:**

Power Doppler showed a good correlation with histological vascular area (Spearman r - 0.73) and angiogenic factors such as vascular endothelial growth factor- A (VEGF-A), Angiopoietin 2 and Tie-2. In addition, lymphangiogenic factors such as VEGF-C and VEGF-R3 correlated well with US assessment of synovitis. A significant correlation was also found between power Doppler and synovial thickness, pro-inflammatory cytokines and sub-lining macrophage infiltrate. Within the supra-patella pouch there was no significant difference in US findings, gene expression or inflammatory cell infiltrate between any regions of synovium biopsied.

**Conclusion:**

Ultrasound assessment of synovial tissue faithfully reflects synovial vascularity. Both grey scale and power Doppler synovitis in early RA patients correlate with a pro-angiogenic and lymphangiogenic gene expression profile. In early RA both grey scale and power Doppler synovitis are associated with a pro-inflammatory cellular and cytokine profile providing considerable validity in its use as an objective assessment of synovial inflammation in clinical practice.

## Introduction

Neovascularization is defined as the development of new blood vessels from the existing microvascular bed. It involves a number of steps including endothelial cell division, selective degradation of vascular basement membranes, alteration of the surrounding extracellular matrix and endothelial cell migration [1,2]. Furthermore, variation in the expression of angiogenic regulators, such as vascular endothelial growth factor (VEGF) and angiopoietin proteins, helps mediate this process and can be detected in synovial tissue [3–5]. Similarly, pro-inflammatory cytokines, such as TNF-α, IL-1 and IL-6, are thought to have a direct influence on new vessel formation within the synovium via VEGF-dependent pathways [6]. Thus a concert of angiogenic factors is at work within the rheumatoid synovium to promote new vessel formation. This is an early, critical aspect to synovial pannus formation and facilitates the perpetuation of the inflammatory process within synovial joints [7]. In addition, lymphatic vessel formation (lymphangiogenesis) is also an important factor for normal tissue homeostasis and can be detected in rheumatoid synovial tissue. Lymphatic vascular networks have previously been described in both patients and animal models of arthritis with VEGF-C, and its ligand VEGF-R3, appearing to promote sprouting of lymphatic vessels in a similar fashion to neoangiogenesis [8–10].

Ultrasound imaging (US) of synovial joints provides an objective assessment of synovial hypertrophy, referred to as grey-scale synovitis, and vascular flow within the synovium, power Doppler signal (PDS). Imaging of this network of new vessels within hypertrophied synovial tissue is purported to provide indirect evidence of tissue inflammation. Previous authors have found correlation between PDS and both macroscopic and microscopic synovial vascularity assessments [11–15]. Most recently, Anderson *et al*. demonstrated that Doppler colour fraction was association with immunohistochemical staining for von Willebrand factor [15]. However, the relationship between US measures of synovitis and synovial tissue angiogenic factors, inflammatory cytokines and vessel density has not been previously explored. The aims of this study were to first examine the variability of ultrasonographic measures of synovitis and synovial vascularity within the knee joint of patients with early rheumatoid arthritis: second, to examine the relationship between histological vascular density and power Doppler signal assessment, and finally, using gene expression, test correlation of both synovial hypertrophy and power Doppler signal with vascular mediators of angiogenesis, lymphangiogenesis and pro-inflammatory cytokines.

## Methods

### Patients

Twelve patients with early rheumatoid arthritis were recruited as part of an ongoing study in patients with early inflammatory arthritis. Disease duration was considered to be from reported symptom onset. All patients were recruited from the early arthritis clinic within the department of rheumatology, Barts Health NHS Trust. All patients had a clinically involved knee joint and fulfilled the American College of Rheumatology (AC) diagnostic criteria (1987) for rheumatoid arthritis. Patients were treatment-naive prior to all assessment and received a clinical assessment, US examination and US-guided synovial biopsy from the supra-patella pouch (SPP). All procedures were performed following written informed consent and were approved by Kings College hospital, London, ethics committee (REC 05/Q0703/198).

### Ultrasound-guided synovial biopsy technique

US-guided synovial biopsy and standard US images were performed using a GE Logic 9 ultrasound machine with a two-dimensional M12L transducer as previously described [16]. Using a 14G Quick-Core® Biopsy Needle (Cook medical, Limerick, Ireland), tissue was harvested from the medial, middle and lateral aspects of the supra-patella pouch. The needle biopsy was repeatedly positioned in each of the three segments and the US probe used to guide the throw of the needle into the correct anatomical position. A minimum of six samples were retrieved from each region of interest (ROI) (typically 6 to 10 samples). Samples were retrieved from each region and separately processed for immunohistochemistry and RNA extraction.

### Ultrasound score

US images of the knee and supra-patella pouch (SPP) were recorded by a single operator (MD) and analysed by two independent examiners (SK, NN) blinded to the clinical and histological data. Three standardised, 3-second cine loops, were recorded pre-biopsy from i) the midline SPP pouch ii) the medial SPP pouch iii) the lateral SPP pouch corresponding to region of synovial tissue sampling with the knee in 30 degrees of flexion. A single sonographer acquired all images. The area demonstrating the maximum Doppler and synovial thickness within each third of the SPP was selected. Training was provided alongside an atlas of probe positions to guide image acquisition. The superior border of the patella was visualized prior to image acquisition. The medial corner of the upper border of the patella corresponding to the base of the medial US image and the lateral corner similarly corresponding to the base of the lateral US image. The midline image included the quadriceps tendon as it inserts into the patella with visualization of the pre-patella fat pad lying superior to the SPP. Synovial tissue was defined as hypoechoic non-compressible intra-articular tissue. Power Doppler settings were adjusted to the lowest permissible pulse repetition frequency to maximize sensitivity. Maximum colour gain was used without creating artefact noise. A standard depth of 3.8 cm was used for each image acquisition. Quantitative analysis of the recorded cine loops was performed using Image J (NIH, [17]. 1997 to 2008) non-proprietary freeware. A dedicated macro was used. The use of a cine loop allows selection of the maximal Doppler signal in that particular ROI given the variations with blood flow with cardiac output. The software facilitates manual drawing of the outline of the synovium within the SPP. Care was taken to isolate synovial fluid from the ROI. The output of the software provides information on the image selected and corresponding pixel count for both synovial area and Doppler signal. Quantitative measurements provided included synovial area quantitative score (SQuant) and power Doppler area quantitative score (PQuant), high-intensity power Doppler signal (representing high-velocity blood flow) over a predetermined colour threshold (PDHi) and a ratio of synovial to Doppler area (PQuant/SQuant). A qualitative score was applied to both the grey-scale synovial thickness (synovial semiquantitative score, SSS) and total power Doppler vascular signal (power Doppler semiquantitative score, PSS) for each region of the SPP on an ordinal scale of 0 to 3. Images were scored quantitatively and qualitatively by two experienced readers (NN and SK) with good agreement (grey-scale synovial thickness intraclass correlation coefficient (ICC) 0.87, power Doppler ICC 0.96).

### Synovial tissue processing

Each synovial specimen was divided into two parts: one was formalin fixed and paraffin embedded for immunohistologic analysis and the second was stored in RNA later (AMBION Life Technologies Ltd, UK) at −80°C until RNA extraction and quantitative PCR analysis.

### Immunohistochemistry

Paraffin-embedded tissue specimens were cut in consecutive 3-μm-thick sections. Haematoxylin and eosin stain was performed to ensure tissue morphology was preserved: only tissue sections with an intact lining layer were used and tissue was graded according to a previously reported scoring system (Krenn synovitis score) [18]. Immunohistochemistry-stained specimens for T-cells, macrophages and plasma cells (CD3, CD68 and CD138 respectively) are scored on a 5-point scale of 0 to 4 depending on the increasing number of positively stained cells calibrated against a standardised atlas [19].

To quantify vascular density in the synovial tissue in terms of both number and size of vessel profiles, double immunofluorescent staining for factor VIII and α smooth muscle actin (α-SMA) was used. The following primary antibodies were used for immunofluorescence analysis: mouse anti human factor VIII (clone F8/86; Dako, Glostrup, Denmark), Cy3 conjugated mouse anti α-SMA (clone 1A4; Sigma-Aldrich, St Louis, MO, USA). The appropriate secondary antibody for factor VIII was obtained from Invitrogen, Paisley, UK. As negative control irrelevant isotype-matched antibodies (Dako, Glostrup, Denmark) were used. Briefly, antigen retrieval was performed by heating for 35 minutes at 95°C in Dako Target Retrieval Solution. Sections when then washed in Tris buffered saline, incubated with protein block (Dako) and then in the first primary antibody for one hour (mouse anti human factor-VIII using antibody dilution of 1:50). After washing, the appropriate Alexa-488 conjugated secondary antibody was applied for 30 minutes; sections were then washed and the directly conjugated second primary antibody, α-SMA, was added at dilution of 1:200. Synovial tissue was counterstained with 4’-6-diamidino-2-phenylindole (DAPI) and mounted with anti-fade mounting medium. To obtain a more representative assessment of synovial vascularity all available synovial specimens were cut and stained at two different cutting levels 50 μm apart. Sections without a defined synovial lining, or with folded areas after staining, were omitted from the analysis. Assessors of histological scores and vascular area assessments were blinded to patient characteristics and US findings.

### Digital image analysis for synovial vascularity evaluation

**Figure 1:**
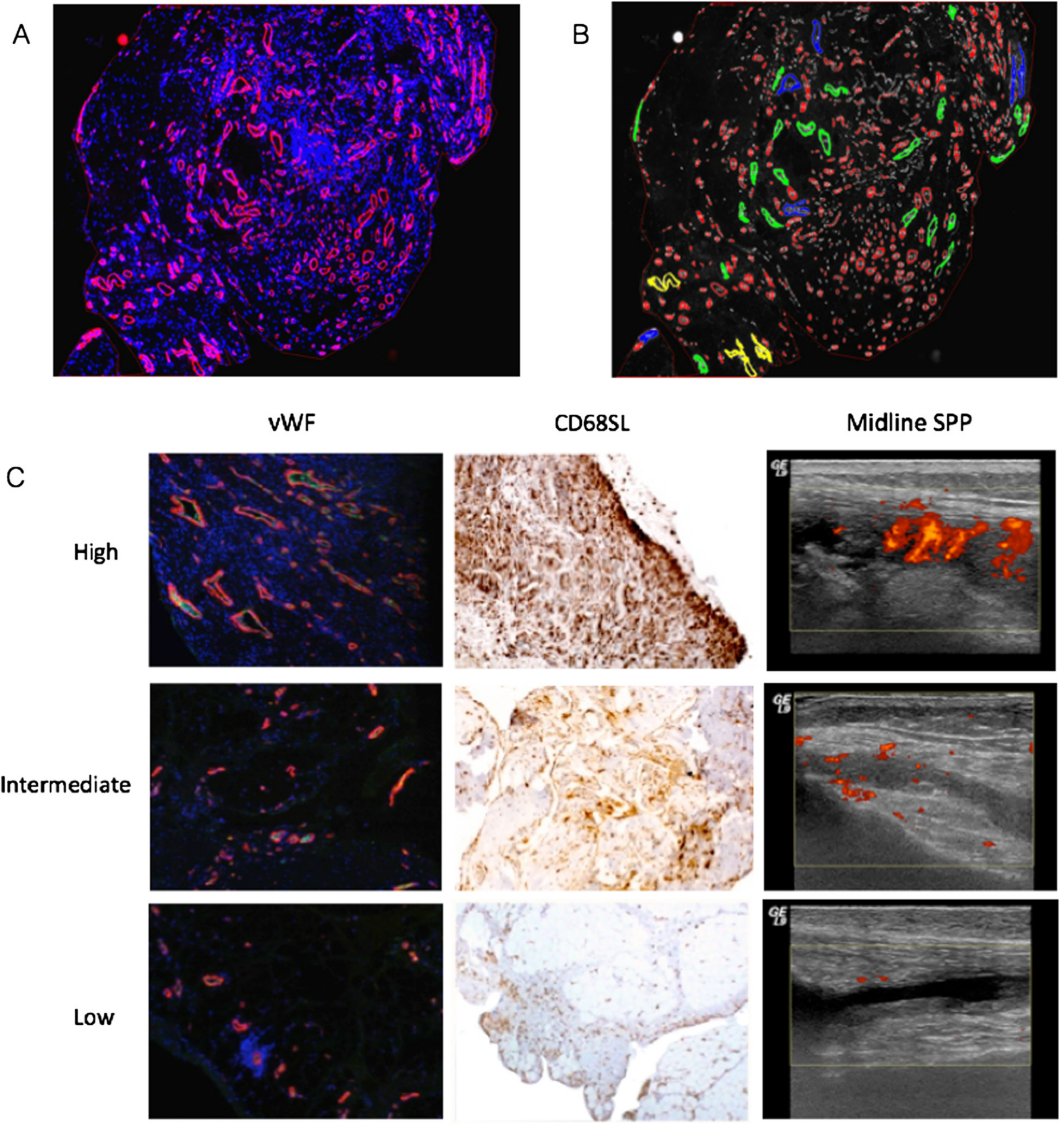
**Synovial tissue stained with factor VIII demonstrating vessels and gated digital analysis for different sized vessels. (A)** Digital image of immunofluorescent-stained synovial blood vessels at 20 times magnification (Olympus BX60 microscope). Synovial tissue with factor VIII staining indicating vessel wall fluorescence (pink). **(B)** Division of vessel size using digital imaging analysis based on specified generated gates with colour differentiation: blue/green, large; red, medium; grey, small. **(C)** Corresponding vascular histology stained with vWF - von willebrand factor, CD68 immunohistochemistry and knee power Doppler images for the midline supra-patella pouch (SPP). vWF and CD68 staining at 10 times magnification (Olympus BX60 microscope). Ultrasound (US) images from GE logiq 9 machine with 12 MHz probe. High, intermediate and low levels of vascularity and inflammation indicated.

One observer, blinded to patients and clinical data, performed both image acquisition and subsequent image analysis for all the sections. Digital images of immunofluorescent-stained synovial blood vessels, were acquired at 20 times magnification on a fully automated Olympus BX61 microscope, captured using a video camera and then digitized using Cell P image analysis software. Each acquisition was performed in one single session for each vascular marker, factor-VIII and α-SMA, respectively, with fixed variable as derived from the calibration. The obtained factor-VIII and α-SMA stained images were saved in jpeg format and used for the subsequent digital images analysis. A selected area was defined as the ROI for both blood vessel density evaluation and digital vessel size analysis. The vascular objects greater than 10 μm in diameter were quantified within the selected ROI, using Cell P analysis software and expressed as blood vessel numerical density (number of vessels per square millimeter, BV/mm^2^) and vessel fractional area (synovial vascular area, SVA/mm^2^), respectively (Figure 1A). Vessels were sub-catergorised using a unique colour based on blood vessel area (Figure 1B). Vascular density, cellular markers of inflammation and US parameters were assessed for each region within the SPP (Figure 1C).

### Quantitative (QT)-PCR of endothelial-specific genes

Total RNA was extracted from the remaining portion of synovial tissue stored in RNA later, using the RNeasy Mini Kit (Qiagen, Chatsworth, CA, USA), with on-column DNase I digestion to avoid genomic DNA contamination. Seven of the thirteen patients had additional tissue available for RNA extraction. cDNA was generated from 1 ug of RNA using the Thermoscript RT-PCR System (Invitrogen, San Diego, CA, USA). QT-PCR was performed to detect mRNA expression levels of Ang-1, Ang 2, VEGF-A, VEGF-C Tie-2 and VEGFR3. The RT-PCR was run in triplicate with an equal loading of 20 ng of cDNA/well. Results were analysed after 40 cycles of amplification using the ABI PRISM 7900HT Sequence Detection System Version 3. Relative quantification was measured using the comparative Ct (threshold cycle) method. cDNA from human placenta was used as a positive control.

### Statistics

Statistical analysis was performed with SPSS version 16. Demographic characteristics of patients are described with mean and interquartile range. Correlation between variables were expressed by Spearman's rho test. Multiple linear regression was performed to analyse the relationship between US parameters and vascular area, immunohistochemistry and gene expression. *P*-values <0.05 were taken as statistically significant.

## Results

### Patient demographics

Twelve patients (nine female, three male) with a mean age of 47 (IQR 37 to 57) years were biopsied. All patients had symptoms of <12 months duration with an average of 7 months from symptom onset to recruitment. Of the patients, 5 (42%) were seropositive for rheumatoid factor with 4 patients (33%) being positive for anti-cyclic citrullinated peptide (anti-CCP) antibodies at presentation. The median disease activity score in 28 joints (DAS28) was 5.41 (IQR 4.46 to 6.34) (Table 1).

**Table 1:** **Patient demographics (n = 12)**

**Variable**	**Value**
**Gender**	
Female	9 (75)
Male	3 (25)
**Age,** **years, median (IQR)**	47 (37 to 57)
**Disease duration, months, median (IQR)**	7.2 (4.9 to 9.5)
**Serology**	
Rheumatoid factor	5 (41.6)
Anti- cyclic citrullinated peptide	4 (33.3)
**Disease activity score in 28 joints (ESR)**	5.41 (4.46 to 6.34)
**Inflammatory markers**	
Erythrocyte sedimentation rate	43 (31 to 55)
C-reactive protein	23 (18 to 28)
**Non-steroidal anti-inflammatory drug use**	4 (33.3)

All patients were recruited form an early arthritis clinic with symptom onset <1 year and mean disease activity score in 28 joints of 5.41. Disease duration taken from the onset of initial symptoms: 42% of patients were rheumatoid factor-positive, 33% were anti-cyclic citrullinated peptide-positive. A third of patients were receiving anti-inflammatory medication prior to recruitment. Numbers are reported with percentage, otherwise, mean and IQR are given where specified.

### Vascularity, gene expression and ultrasound parameters are stable within the supra-patella pouch

**Figure 2:**
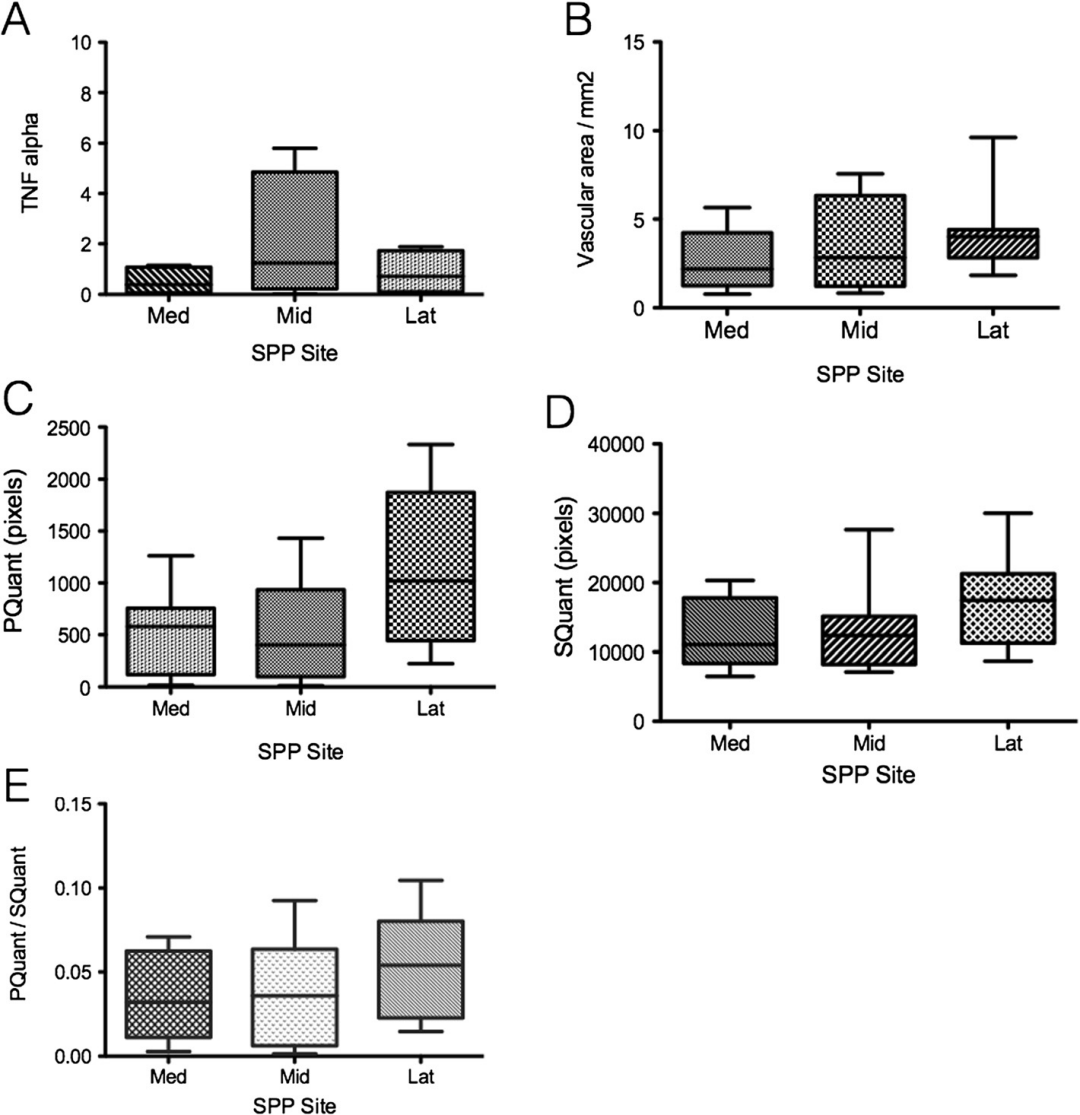
**There was no significant variation of ultrasound, vascular area and gene expression between each region of the supra-patella pouch (SPP).** **(A)** TNF-α expression by each region of interest within the SPP (Kruskal-Wallis test, *P* = 0.31). **(B)** Quantitative power Doppler area (PQuant) (pixels) by each region of interest within the SPP (Kruskal-Wallis test, *P* = 0.27). **(C)** Synovial thickness quantitative area (SQuant) (pixels) by each region of interest within the SPP (Kruskal-Wallis test, *P* = 0.19). **(D)** Synovial vascular area/mm2 by each region of interest within the SPP (Kruskal-Wallis test, *P* = 0.09). **(E)** PQuant/SQuant ratio by each region of interest within the SPP (Kruskal-Wallis test, *P* = 0.44).

The three areas of the SPP were analysed for angiogenic gene expression, vascular density and US parameters. Within the SPP there was no significant variation in TNF-α expression (Kruskal-Wallis test, *P* = 0.31) (Figure 2A). There was no significant difference found in the gene expression profile for any other angiogenic/lymphangiogenic factors or pro-inflammatory cytokines examined. In addition, there was no significant difference in the vascular area measurement in each region, although there was a tendency for the lateral compartment to have slightly greater values than the medial and middle compartments (Figure 2B). No difference was detected in the US parameters within the SPP pouch although again, higher levels of PQuant and PSS were noted in the lateral compartment along with higher levels of SQuant and SSS (Figure 2C and D). The ratio of PQuant/SQuant remained stable throughout the three regions of interest with no significant difference detected between regions (Figure 2E).

### Grey scale and power Doppler synovitis correlate with angiogenic, lymphangiogenic gene expression and sublining macrophage infiltrate

**Figure 3:**
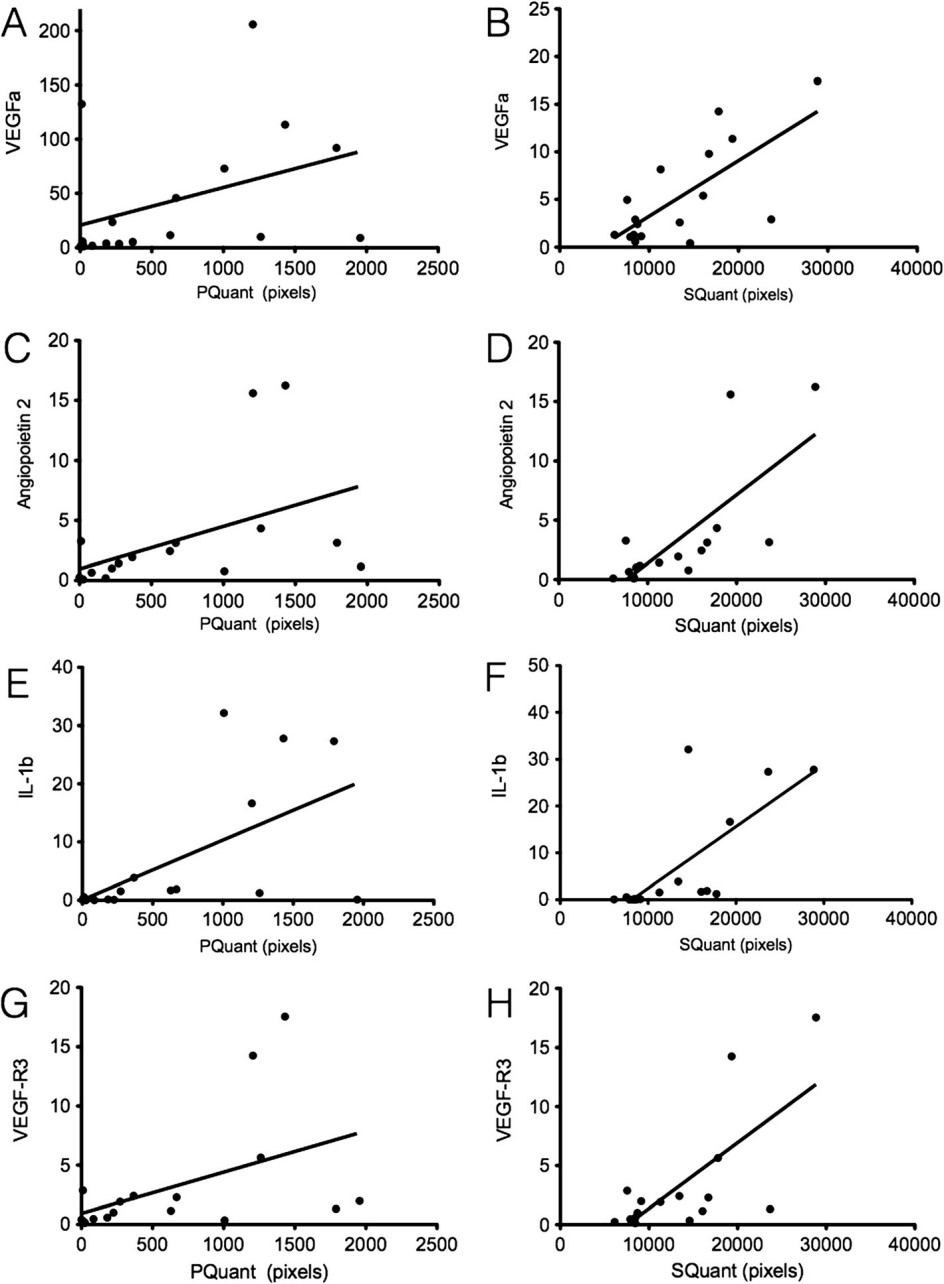
**Quantitative power Doppler area (PQuant) and synovial thickness quantitative area (SQuant) correlate with angiogenic and lymphangiogenic factors.** Graphical representation of relationship of PQuant and SQuant (measured in pixels) with vascular endothelial growth factor (VEGF)α, Angiopoietin 2, IL-1β and VEGF-R3. Regression line and 95% CI shown in each graph. **(A)** Correlation between PQuant and VEGFa on the y-axis (Spearman *r* = 0.52). **(B)** PQuant correlated with Angiopoietin 2 (Spearman *r* = 0.61). **(C)** PQuant correlated with IL-1β (Spearman *r* = 0.68). **(D)** PQuant correlated with VEGF-R3 (Spearman *r* = 0.61). **(E)** SQuant correlated with VEGFα on the y-axis (Spearman *r* = 0.56). **(F)** SQuant correlated with Angiopoietin 2, (Spearman *r* = 0.74). **(G)** SQuant correlated with IL-1β (Spearman *r* = 0.81). **(H)** SQuant correlated with VEGF-R3 (Spearman *r* = 0.61).

PQuant correlated well with TNF-α, IL-1β and IL-6 gene expression (Spearmans rho 0.61, 0.69 and 0.59, respectively). Good correlation was also demonstrated with angiogenic factors such as VEGF-A and VEGF-R3 (Figure 3A-D). SQuant correlated significantly with pro-inflammatory cytokine expression and angiogenic factors such as VEGF-A, Angiopoietin 2 and Tie 2 (Figure 3E-H). With respect to lymphangiogenesis, significant correlation was confirmed with synovial thickness area and VEGF-R3, VEGF-C, and Podoplanin, a lymphatic endothelial marker (Spearmans rho 0.61, 0.62 and 0.71 respectively). Modest correlation was also demonstrated with CD68SL macrophage infiltration and quantitative US assessments (PQuant, SQuant, PDHi). Table 2 provides an overview of the correlation matrix of US parameters, angiogenic, lymphangiogenic factors and cellular mediators of inflammation.

**Table 2:** **Correlation matrix of ultrasound parameters with angiogenic, lymphangiogenic cytokine expression and cellular infiltration**

**US parameter**	**PSS**	**SQuant**	**PQuant**	**PDHi**	**PQuant/SQuant**
TNF-α	0.46*	0.74*	0.61*	0.60*	0.59*
IL-6	0.30	0.69*	0.59*	0.56*	0.57*
IL-1β	0.47*	0.81*	0.68*	0.64*	0.67*
Podoplanin	0.19	0.71*	0.43	0.15	0.32
Angiopoietin 1	0.4	0.37	0.22	0.11	0.23
Angiopoietin 2	0.5	0.74*	0.61*	0.46	0.56*
VEGF-R3	0.50*	0.61*	0.55*	0.36	0.47
VEGF-C	0.2	0.62*	0.39	0.14	0.29
VEGF-A	0.5	0.56*	0.52*	0.48*	0.54*
Tie-2	0.3	0.51*	0.44	0.19	0.34
Synovitis score	0.25	0.18	0.27	0.48*	0.31
CD68SL	0.29	0.35*	0.40*	0.51*	0.40*
CD68L	0.12	0.46*	0.18	0.33	0.09
CD20	0.02	0.24	0.25	0.38*	0.22

Correlation was analysed using Spearman's rho. *Significant values (*P* <0.05). SQuant correlated with the majority of pro-inflammatory cytokines, angiogenic, lymphangiogenic gene expression and macrophage lining and sublining infiltration. PSS, power Doppler semiquantitative score; SQuant, quantitative synovial thickness area; PQuant, quantitative power Doppler area; PDHi, quantitative thresholded power Doppler area; VEGF, vascular endothelial growth factor, Tie, Tyrosine kinase (predominant endothelial cell expression), CD68SL, macrophage marker (synovial sublining layer), CD68L, macrophage marker (synovial lining layer).

### Synovial vascular area is a predictor of power Doppler signal

**Figure 4:**
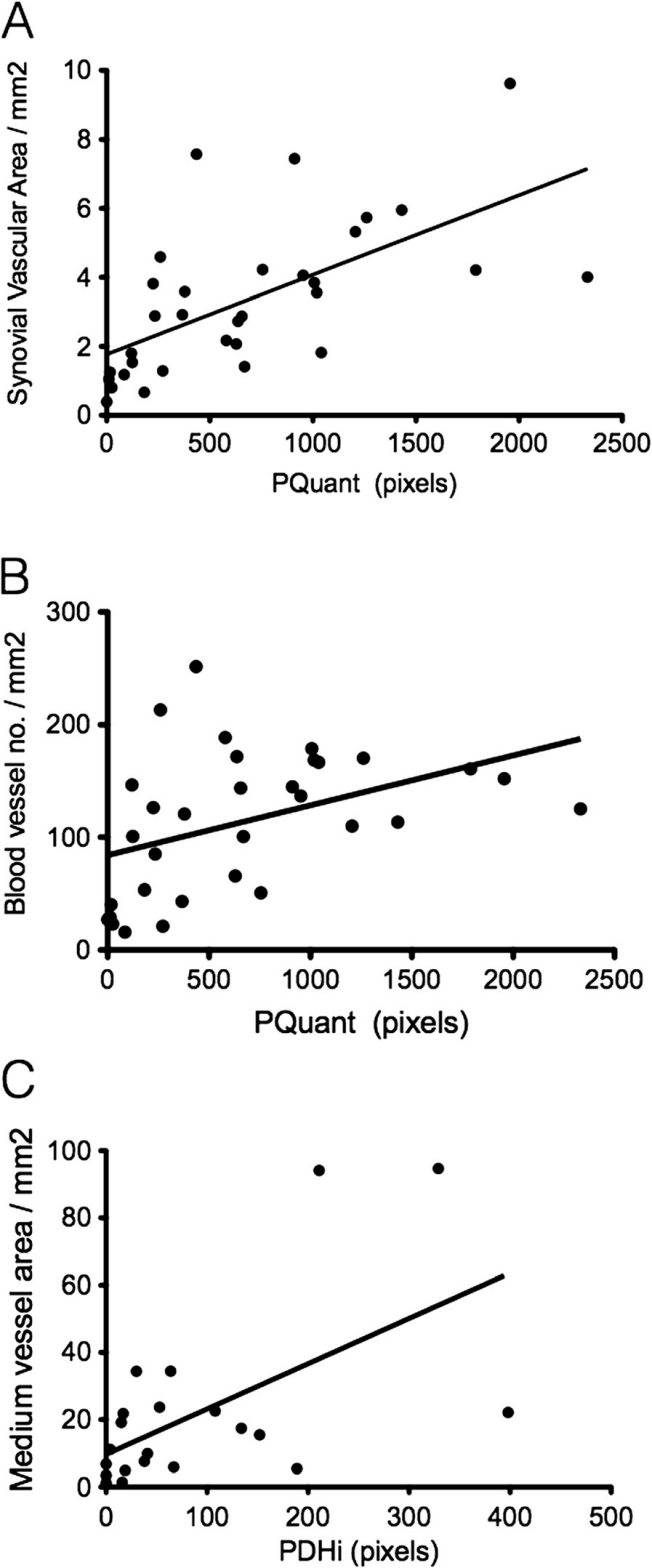
**Synovial vascular area predicts Doppler signal within the knee joint. (A)** Qualitative Doppler assessment (PQuant) correlates with synovial vascular density within the supra-patella pouch (Spearman *r* 0.73). **(B)** PQuant correlates with blood vessel density (Spearman *r* 0.42). **(C)** Thresholded Doppler signal for high intensity signal (PDHi) correlates with medium-sized vessel vascular area (Spearman *r* 0.56).

Quantitative and semiquantitative US parameters were correlated with synovial vascular density and blood vessel number density. PQuant correlated with SQuant/mm^2^ and BV/mm^2^ (Spearman's rho 0.73 and 0.53, respectively) (Figure 4A, B). All US parameters positively correlated with both histological assessments of synovial vascularity except for BV/mm^2^ which did not correlate significantly with the semiquantitative PSS. Linear regression showed a positive relationship between quantitative power Doppler area assessment and histological synovial vascular area/mm^2^ (*r*^2^ 0.49). Division and analysis of the synovial vascular area by size of vessel demonstrated that medium-sized vessels contributed most to the overall vascular area (79%) within each tissue specimen with small and large vessels contributing in 9.5% and 11.5%, respectively. A significant correlation between the PDHi and medium-sized vessels overall area was demonstrated (Spearman's rho 0.55) (Figure 4C).

## Discussion

For patients with suspected inflammatory arthritis undergoing US examination, the detection of grey-scale synovial thickening and power Doppler signal within synovial joints is an important aspect of the disease assessment. An appreciation of the ultrasonographic histopathological correlates is essential to the interpretation of these findings in the clinical setting. To minimize the influence of treatment effects in this patient population, patients naive to therapy (including corticosteroids and disease modifying drugs) were recruited. All had active rheumatoid disease with a moderate-to-high DAS 28 score prior to biopsy and a knee joint which was deemed clinically inflamed.

In this study, histologically assessed synovial vascular area was an independent predictor of ultrasound assessment of power Doppler signal as assessed by PQuant and PDHi. Assessing the relationship of PQuant to synovial vascularity in this study differed from previous reports. Synovial vascularity has previously been assessed macroscopically, with immunohistochemistry staining of factor VIII and by a semiquantitative ordinal scale [11–13,15,20]. In this study we favoured a quantitative approach to tissue assessment with the vascular area being scored using digital imaging analysis. This has a number of advantages including scoring standardisation and providing a fractional vascular area assessment in addition to a measure of the vessel number and density. In addition to vascular density correlation was also detected between power Doppler signal and pro-inflammatory cytokines (IL-1β and TNF-α). This may suggest that other factors exert influence over the generation of Doppler signal in addition to the available synovial vascular networks. It is likely that these vasoactive cytokines contribute by exerting a degree of control over the afferent vascular tone, modifying the local blood flow. The correlation of other angiogenic factors (such as VEGF-A, Tie2 and Ang 2, but not with Ang 1) with both synovial thickness and power Doppler, may indicate a profile of synovial tissue primed for neovascularisation. Upregulation of VEGF-A and Ang 2 in this context may be an important factor in neovascularization within the rheumatoid joint. Similarly, over expression of VEGF-A and Ang 2 has a positive effect on tumour growth while over-expression of Ang 1 reduces tumour expansion [21,22]. We also demonstrated that expression of lymphangiogenic factors correlated with ultrasound parameters and in particular, synovial area assessment. Lymphangiognesis is an important process in tissue development and regulation with VEGF-C and its receptor VEGF-R3 are known to be important factors in this process [8,23]. The association with the quantitative synovial area score may suggest that this is seen within an expanding, inflamed synovium. However, a report from Polzer *et al*. suggests that lymphangiogenesis is encouraged following treatment with anti-TNF-α therapy with a greater expression of lymphatic vessels in synovial tissue when examining pre- and post-treatment synovial tissue [10]. The expression of VEGF-C and VEGF-R3, however, were not specifically commented upon in this study. The angiogenic/lymphangiogenic profile data presented here, however, are cross-sectional, thus limiting any conclusions about the regulation of these factors following treatment or any change in their relationship with US assessment.

Histological parameters and angiogenic gene expression demonstrated variation within the SPP. Furthermore, the US parameters did reveal a higher average signal in the lateral compartment of the joint. The small number of patients in this study, and subsequent limited gene expression data available in 7 out of 12 patients, means that a conclusion about the variation in gene expression, US parameters and histology throughout the joint space is problematic. Although none of these comparisons were statistically significant, the small number of patients within this study is likely to be underpowered to detect a real difference.

Synovial hypertrophy, as assessed by US, consists of a number of cellular elements (resident and migratory), interstitial fluid, connective tissue and a vascular network. This study shows a positive correlation with both cellular elements such as CD68SL macrophages, pro-inflammatory cytokines, and both angiogenic and lymphangiogenic factors. This cocktail of cellular infiltration and cytokines promotes an inflammatory environment responsible for many of the changes taking placed within the joint.

## Conclusion

In this study of patients with early rheumatoid arthritis, we demonstrate a clear relationship between power Doppler signal, histologically determined synovial vascular area and angiogenic gene expression. Grey-scale assessment of synovial thickness also correlated with cellular markers of synovial inflammation and pro-inflammatory cytokines. Therefore, the assessment of synovial joint inflammation in early arthritis, by ultrasound, has considerable validity. How these relationships alter and potentially evolve following therapeutic intervention can only be dissected further in a prospective fashion.

## Abbreviations

Ang: angiopoietin
α-SMA: α smooth muscle actin
BV/mm^2^: number of vessels per square millimetre
CCP: cyclic citrullinated peptide
CRP: C-reactive protein
DAS: disease activity score
DMARD: disease-modifying antirheumatic drug
ESR: erythrocyte sedimentation rate
HAQ: health assessment questionnaire
ICC: intraclass correlation coefficient
IL: interleukin
OMERACT: Outcome Measures in Rheumatology Clinical Trials
PDHi: power Doppler threshold area of high signal
PDS: power Doppler signal
PQuant: power Doppler quantitative area
PSS: power Doppler semi-quantitative score
QT-PCR: quantitative PCR
RA: rheumatoid arthritis
RF: rheumatoid factor
ROI: region of interest
RT-PCR: real-time polymerase chain reaction
SJC: swollen joint count
SPP: suprapatella pouch
SQuant: synovial thickness quantitative area
SSS: synovial semi-quantitative score
SVA/mm^2^: synovial vascular area
Tie: tyrosine kinase
TJC: tender joint count
TNF: tumour necrosis factor
US: ultrasound
VEGF: vascular endothelial growth factor
